# Podocyte Injury in Diabetic Kidney Disease: A Focus on Mitochondrial Dysfunction

**DOI:** 10.3389/fcell.2022.832887

**Published:** 2022-03-07

**Authors:** Simeng Liu, Yanggang Yuan, Yi Xue, Changying Xing, Bo Zhang

**Affiliations:** ^1^ Department of Nephrology, The First Affiliated Hospital of Nanjing Medical University, Nanjing Medical University, Nanjing, China; ^2^ Suzhou Hospital of Integrated Traditional Chinese and Western Medicine, Suzhou, China; ^3^ Department of Nephrology, Pukou Branch of JiangSu Province Hospital (Nanjing Pukou Central Hospital), Nanjing, China

**Keywords:** podocytes, diabetic kidney disease, mitochondrial dysfunction, therapeutic strategies, injury

## Abstract

Podocytes are a crucial cellular component in maintaining the glomerular filtration barrier, and their injury is the major determinant in the development of albuminuria and diabetic kidney disease (DKD). Podocytes are rich in mitochondria and heavily dependent on them for energy to maintain normal functions. Emerging evidence suggests that mitochondrial dysfunction is a key driver in the pathogenesis of podocyte injury in DKD. Impairment of mitochondrial function results in an energy crisis, oxidative stress, inflammation, and cell death. In this review, we summarize the recent advances in the molecular mechanisms that cause mitochondrial damage and illustrate the impact of mitochondrial injury on podocytes. The related mitochondrial pathways involved in podocyte injury in DKD include mitochondrial dynamics and mitophagy, mitochondrial biogenesis, mitochondrial oxidative phosphorylation and oxidative stress, and mitochondrial protein quality control. Furthermore, we discuss the role of mitochondria-associated membranes (MAMs) formation, which is intimately linked with mitochondrial function in podocytes. Finally, we examine the experimental evidence exploring the targeting of podocyte mitochondrial function for treating DKD and conclude with a discussion of potential directions for future research in the field of mitochondrial dysfunction in podocytes in DKD.

## Introduction

Diabetic kidney disease (DKD) is the leading cause of end-stage renal disease (ESRD), and it affects nearly 30–40% of patients with diabetes (Alicic et al., 2017). Although remarkable progress in drug therapy has reduced the rate of diabetes-related cardiovascular complications, the incidence of DKD and renal failure has continued to rise (Gregg et al., 2014). The principal feature of DKD is an abnormality of the glomerular filtration barrier (GFB), leading to the leakage of protein (proteinuria), metabolites and ions into the urine. Proteinuria simultaneously acts as a major, independent risk factor for the progression of DKD to ESRD. Podocytes form the outer part and ensure the mechanical stability of the GFB, therefore preventing protein loss into the urine. Podocyte dysfunction is one of the earliest glomerular morphologic changes and it plays a key role in DKD progression (Wang et al., 2012; Reidy et al., 2014; Qi et al., 2017).

Mitochondria, the main energy-producing organelles, play a central role in cell survival and death signalling. Mitochondria respond to pathophysiologic cues by altering their content, fusion, fission, mitophagy, and the unfolded protein response (UPR). Hyperglycemia is the most predominant clinical abnormality in diabetes, and it has been viewed as one of the leading risk factors for the pathogenesis of DKD. High glucose (HG) toxicity is mediated by many abnormal glucose metabolic pathways or signalling pathways that can induce reactive oxygen species (ROS) overproduction and mitochondrial damage (Qi et al., 2017). These factors may in turn cause oxidative stress, inflammation, and cell apoptosis. Indirect evidence for mitochondrial dysfunction of podocytes involved in DKD from diabetic models has been accumulating (Susztak et al., 2006; Wang et al., 2012; Ayanga et al., 2016; Qin et al., 2020; Wei et al., 2020), and studies have directly observed mitochondrial dysfunction in clinical samples from patients with DKD (Sharma et al., 2013; Czajka et al., 2015). A variety of mitochondrial dysfunction pathways have been identified as the main molecular causes of podocyte injury, such as elevated mitochondrial ROS production (Jha et al., 2016), imbalanced mitochondrial dynamics (Ayanga et al., 2016) and decreased mitochondrial biogenesis (Bhargava and Schnellmann 2017; Qin et al., 2020). Notably, mitochondrial dysfunction induced by glucose toxicity is usually considered to be an irreversible process owing to the persistence of epigenetic reprogramming (Reidy et al., 2014). For example, Chen *et al.* found persistent differential methylation at several loci over more than 16–17 years in a same cohort (Chen et al., 2016).

Podocytes are terminally differentiated with poor capacity to re-enter the cell cycle and proliferate. Mitochondrial dysfunction is the major contributor to podocyte injury and death, where an abnormal energy supply may lead to irreversible cellular injury (Carney 2015; Arif et al., 2019). Podocytes require a substantial amount of energy to maintain the complex cellular morphology of tertiary foot processes. Mitochondrial DNA mutations could cause podocyte dysfunction and breakdown of the GFB (Heeringa et al., 2011), and data from animal models support this hypothesis (Baek et al., 2018; Widmeier et al., 2020). The above findings support mitochondria involvement in the pathogenesis of podocyte injury, and regulating podocyte energy metabolism by targeting mitochondria may promote podocyte recovery from injury.

**FIGURE 1:**
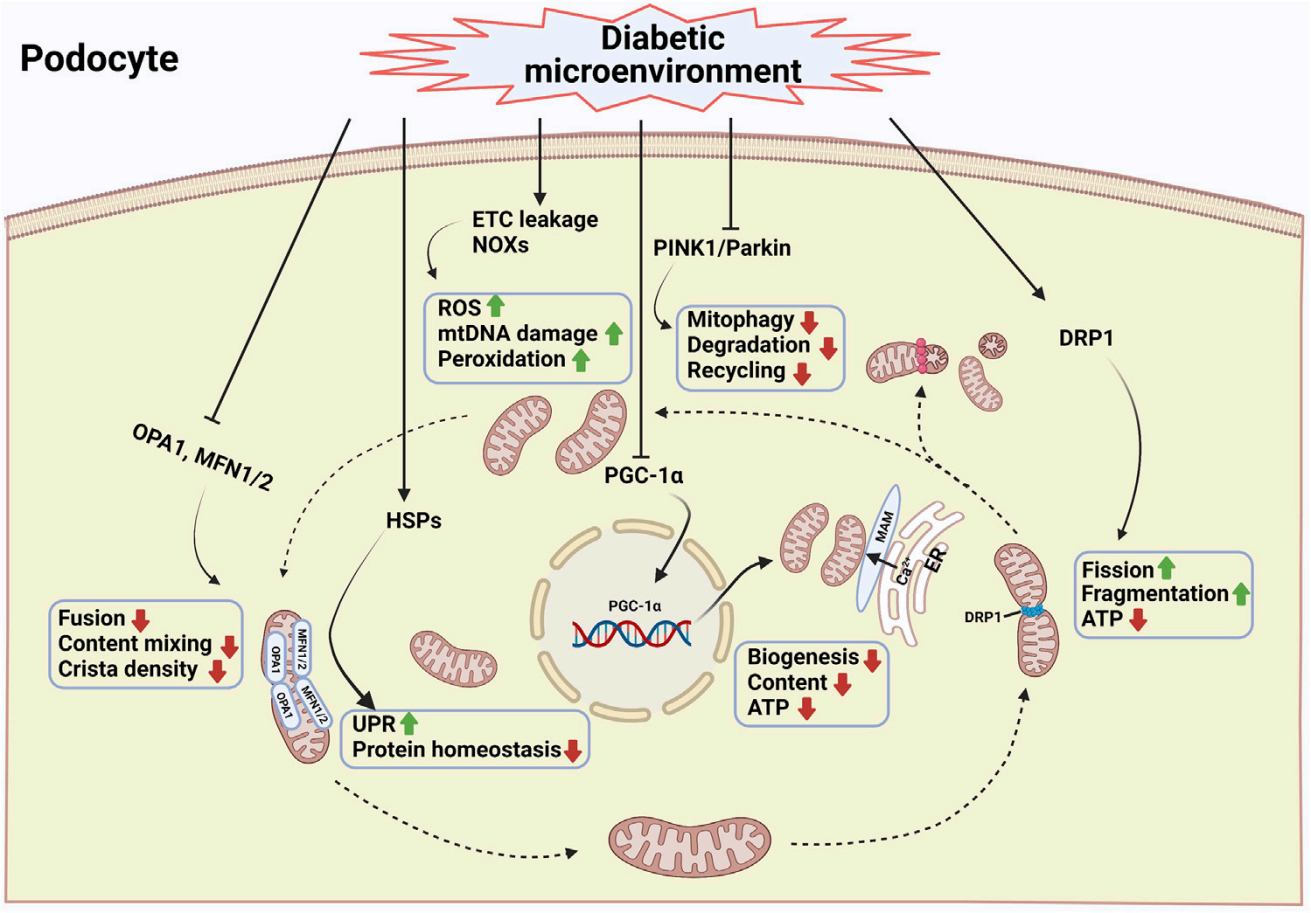
Mitochondrial damage of podocytes during diabetic kidney disease. Mitochondria are highly dynamic organelles that respond to pathophysiologic cues by altering mitochondrial content, fusion, fission, mitophagy, and the unfolded protein response. Fission and fusion complement each other to maintain mitochondrial morphology, whereas mitophagy selectively clears damaged mitochondria from the network (Nisoli et al., 2004). Excessive mitochondrial fission combined with decreased mitochondrial fusion is a prototypical feature of podocytes in diabetic kidney disease (Wang et al., 2012; Ayanga et al., 2016; Qin et al., 2019; Audzeyenka et al., 2021). In addition, the inhibition of mitophagy leads to the lack of a proper mitochondrial turnover in the diabetic kidney (Li et al., 2016; Li W. et al., 2017). Another key feature of mitochondrial dysfunction of podocytes in diabetic kidney disease is the reduced efficiency of mitochondrial biogenesis (Sun et al., 2014; Li S.-Y. et al., 2017; Zhang et al., 2018). Under high glucose condition, intracellular ROS production, mitochondrial DNA damage and protein and lipid peroxidation were enhanced (Tan et al., 2010; Dugan et al., 2013; Coughlan et al., 2016). In addition, mitochondrial protein homeostasis is challenging because of the continuous exposure of mitochondrial proteins to mitochondrial ROS. Mitochondria within a cell cannot exist in isolation. They interact with endoplasmic reticulum via the formation of mitochondrial-associated membranes (MAMs). The disturbance of MAMs leads to abnormal intracellular Ca^2+^ influx, mitochondrial damage, and apoptosis (Inoue et al., 2019). A combination of the above factors resulted in podocyte injury and the progression of diabetic kidney disease. The podocyte mitochondria in diabetic condition is illustrated schematically with blue frame and text. DRP1, dynamin-1-like protein; MFNs, mitofusin proteins 1 and 2; ETC, electron transport chain; HSPs, heat shock proteins; MAM, mitochondria associated ER membrane; NOXs, NADPH oxidases; OPA1, optic atrophy protein 1; PGC-1α, peroxisome proliferator activated receptor γ coactivator-1α; PINK1, PTEN-induced putative kinase protein 1; ROS, reactive oxygen species; UPR, unfolded protein response (Created with BioRender.com).

The regulation of podocyte mitochondrial dysfunction in patients with DKD has been extensively studied in the past few years, but few reviews have thoroughly summarized the progress in this area. In this review, we summarize the latest research progress on the causes of acquired mitochondrial dysfunction in podocytes in DKD (Figure 1). A comprehensive investigation of mitochondrial damage and its potential regulatory mechanisms could provide a deeper understanding of podocyte injury and possible therapeutic options that could have a positive impact on the treatment of DKD.

## Mitochondrial Quality Control and Podocytes Injury in DKD

### Mitochondrial Dynamics and Mitophagy

Mitochondria are dynamic organelles that frequently change their content and distribution within the cell. Dynamic remodelling of mitochondrial networks by fission, fusion and mitophagy promotes the maintenance of cellular function and survival under different physiological conditions. Mitochondrial fission and fusion processes appear to be accompanied by mitophagy. Fission and fusion complement each other to maintain mitochondrial morphology, whereas mitophagy selectively clears damaged mitochondria from the network (Nisoli et al., 2004). Of note, mitochondrial fission is thought to be a central process required for mitochondrial autophagy whereas mitochondrial elongation through fusion inhibits mitophagy. Accordingly, mutations in genes encoding fission and fusion proteins are associated with genetic diseases, highlighting the importance of sustaining mitochondrial dynamics (Sheffer et al., 2016; Gerber et al., 2017).

#### Fission and Fusion

Fission is the division of mitochondria in two by cleavage of the inner mitochondrial membrane (IMM) and outer mitochondrial membrane (OMM) and fusion is the combination of two mitochondria into one by the joining of the OMM and IMM. Fission is necessary to facilitate the autophagic removal of damaged mitochondria. Fission and fusion are complementary processes. Rather than being phenomenological, mitochondrial dynamics also influence mitochondrial functions and energy metabolism in many ways.

Mitochondrial fusion is regulated by several proteins, including mitofusin1 (MFN1) and mitofusin2 (MFN2) located in the OMM and optic atrophy protein 1 (OPA1) located in the IMM (Song et al., 2015). MFN1 is necessary for OMM fusion, whereas OPA1 is important for IMM fusion. Mice deficient in either MFN1 or MFN2 die in utero in midgestation (Chen et al., 2003). The precise role of MFN2 in fusion is not yet clear and it may be involved in the interaction between mitochondria and other organelles [in particular with the endoplasmic reticulum (ER)] (de Brito and Scorrano 2008). Fusion allows for content mixing, thus buffering the effect of damaged mitochondrial DNA, proteins, lipids and metabolites and maintaining normal mitochondrial activity (Youle and van der Bliek 2012; Pickles et al., 2018). MFN1 and MFN2 initiate the fusion process by tethering OMMs of adjacent mitochondria, and external stimuli, such as oxidative stress, can enhance OMM fusion (Shutt et al., 2012). Then, OPA1 completes the fusion process at IMMs. IMM fusion is more complicated than OMM fusion maintaining crista structures of the IMM. Deletion of OPA1 induces mitochondrial fragmentation and also results in decreased cristae density, in which the IMM becomes vesicular (Meeusen et al., 2006).

Mitochondrial fission is primarily driven by dynamin-related protein 1 (Drp1), a gtpase that dynamically associates with the ER and mitochondria (Ji et al., 2017). Drp1 translocates from the cytosol to the surface of the OMM, binding to its receptors in the OMM, including mitochondrial fission factor (MFF) (Otera et al., 2010; Sheng et al., 2019), mitochondrial dynamics proteins 49 and 51 (MID49/51) (Palmer et al., 2013) and mitochondrial fission 1 protein (Fis1) (James et al., 2003). Following this binding, Drp1 oligomerizes to form a constrictive ring around the mitochondrion to physically constrict and sever the mitochondrion (Kalia et al., 2018; Adachi et al., 2020). Drp1 is essential for embryonic development, and genetic knockout of Drp1 in mice is embryonically lethal at approximately embryonic Days 11.5–12 (Wakabayashi et al., 2009). However, cultured mammalian cells can survive without Drp1 and undergo mitochondrial fission *in vitro* (Kraus et al., 2021). Therefore, the functional role of mitochondrial fission is easier to detect *in vivo* studies in which damaged mitochondrial networks impair a variety of cellular biological activities, such as autophagy and apoptosis. Mitochondrial fission is necessary to surveil and isolate damaged mitochondria, which plays a key role in the quality control of the mitochondrial network (Bhargava and Schnellmann 2017). These daughter mitochondria with high membrane potential may recover by fusion (Abrisch et al., 2020) while unbalanced and depolarized daughter mitochondria are degraded through mitophagy to sustain a population of healthy mitochondria (Dikic and Elazar 2018; Kraus et al., 2021).

Defective mitochondrial dynamics have harmful effects on mitochondrial homeostasis and have been closely linked to the pathogenesis of numerous diseases, including cancer, cardiovascular diseases, and CKD (Galloway et al., 2012). Recently, several studies have proposed that excessive mitochondrial fission and enhanced fragmented mitochondria in podocytes are characteristic features of kidney injury before the obvious clinical manifestations of DKD (Wang et al., 2012; Ayanga et al., 2016; Qin et al., 2019). Drp1-specific knockdown in podocytes or pharmacologic inhibition of Drp1 by mitochondrial division inhibitor 1 (Mdivi-1) in diabetic mice confers protection against DKD with decreased albuminuria and improved morphology compared to diabetic control mice (Ayanga et al., 2016). Accordingly, podocytes isolated from Drp1 knockout mice demonstrated more elongated mitochondria and ATP production was restored, unlike in podocytes isolated from wild-type mice. Furthermore, the herbal alkaloid berberine could significantly protect podocytes via inhibiting Drp1-mediated mitochondrial fission and cell apoptosis, suggesting its use as a new therapeutic drug to treat DKD (Qin et al., 2019). Collectively, the data available indicate that mitochondrial fragmentation contributes critically to podocyte injury in DKD. Research is, however, still in an early stage.

Mediators that increase the expression of Drp1 or promote Drp1 translocation to the OMM both contribute to mitochondrial fission (Ayanga et al., 2016; Deng et al., 2020). Drp1 activity and translocation can be affected by posttranslational modifications, such as phosphorylation (Wang et al., 2012; Sabouny and Shutt 2020), *O*-GlcNAcylation (Gawlowski et al., 2012), and sumoylation (Zunino et al., 2007; Braschi et al., 2009). Among these post-translational modifications, Drp1 phosphorylation seems to play a central regulatory role, which can exert either activating or inhibitory effect depending on the specific site modified. Several phosphorylation sites have been identified in Drp1, including Ser-579, Ser-40, Ser-585, Ser-44, Ser-592, Ser-656, Ser-616, Ser-637, and Ser-693 (Qi et al., 2019). Among these sites, both Ser-616 and Ser-637 have been extensively reported in various diseases, while only Ser-637 was deeply examined in podocytes. Drp1 is a cytosolic protein, and phosphorylation at Ser637 of Drp1 promotes Drp1 translocation to mitochondrion to induce fission in response to HG conditions in podocytes (Ayanga et al., 2016). Similarly, activated A-kinase anchoring protein 1 (AKAP1) promotes the phosphorylation of Drp1 at Ser637, which promotes the transposition of Drp1 to the OMM and results in mitochondrial dysfunction events in HG-induced podocyte injury (Chen et al., 2020). However, another study, inconsistent with these findings, found that Drp1 phosphorylation at Ser637 by phosphoprotein kinase A (PKA) inhibits its gtpase activity and inhibits fission (Cribbs and Strack 2007). In contrast, Drp1 dephosphorylation at the same site by the Ca^2+^-dependent phosphatase calcineurin activates Drp1 and promotes fission (Cereghetti et al., 2008). A possible explanation for these conflicting results could be that the effects of Drp1 phosphorylation at this residue are likely cellular context- and external stimulus-dependent (Galvan et al., 2017). Because of the complexity of the posttranslational modification, the external stimuli that trigger this pathway remains largely unknown and needs investigation.

#### Mitophagy

Mitophagy is the best-described form of selective autophagy. It specifically degrades long-lived or damaged mitochondria via the formation of intracellular organelles-mitophagosomes. Mitophagosomes ultimately fuse with lysosomes, finally resulting in content degradation. The half-life of mitochondria is 10–25 days in the human body, and mitophagy serves as a master regulator in the maintenance of the quality of the mitochondrial pool in response to metabolic demand. If mitochondria are damaged beyond repair, mitochondria are eliminated through mitophagy to prevent ROS production, provide raw materials for metabolic needs and contribute to mitochondrial biogenesis. Abnormal or excessive mitophagy has been implicated in numerous human disorders (Palikaras et al., 2018).

Generally, mitophagy is divided into PTEN-induced putative kinase protein 1 (PINK1)/Parkin-dependent or PINK1/Parkin-independent (receptor-mediated) pathways. PINK1/Parkin-dependent mitophagy can be initiated by a loss of mitochondrial membrane potential (MMP), while the activation of the PINK1/Parkin-independent pathway is regulated through receptors that are anchored on the cytosolic surface of the OMM such as BNIP3L, BCL2-L13, and FUNDC1 (Ng et al., 2021). Mitophagy in most cell types is induced by the PINK1/Parkin-mediated pathway. Under physiological conditions, the mitophagy signal protein PINK1 is translocated from the cytosol to the IMM and then degraded by mitochondrial proteasomes (Greene et al., 2012). As PINK1 import is dependent on an intact MMP, mitochondrial damage or depolarization hampers its translocation and results in its accumulation on the OMM (Choi 2020). Then PINK1 recruits the E3 ubiquitin ligase Parkin to the mitochondria and prompts its phosphorylation/activation, which ubiquitinates lysine residues in the N-termini of OMM proteins, thereby targeting the mitochondria for degradation by autophagosomes (Randow and Youle 2014; Choi 2020). Meanwhile, simultaneous phosphorylation of ubiquitin chains by PINK1 might further facilitate Parkin activation and recruitment (Lazarou et al., 2015).

Autophagy is well known to be exacerbated in podocytes in DKD (Liu et al., 2019), and relatively few studies have explored the effect of mitophagy on podocytes in DKD. Mitophagy is considered as a defense mechanism under pathological conditions. Thus, we can infer that mitophagy is induced to ensure mitochondrial quality control by clearing damaged mitochondria during the initial stage of DKD. However, as the disease progresses, the increased number of damaged mitochondria might exceed the eliminated capacity of mitophagy, or mitophagy might also become impaired, then the apoptotic pathway is activated to minimize tissue damage. It has been demonstrated that HG accelerates mitochondrial dysfunction and podocyte apoptosis by inhibiting mitophagy activity (Li et al., 2016; Li W. et al., 2017). Overexpression of forkhead-box class O1 (FOXO1) in podocytes activates PINK1/parkin-dependent mitophagy, which degrades dysfunctional mitochondria and alleviates podocyte injury in diabetic mice and cultured podocytes, supporting the hypothesis of an important role of FOXO1 in the regulation of mitophagy in podocytes (Li W. et al., 2017). Progranulin (PGRN), an autocrine growth factor, has been known to be involved in the development and/or progression of various inflammatory diseases including renal ischemia/reperfusion injury and diabetic complications (Xu et al., 2015; Zhou et al., 2015; Choi et al., 2020). In diabetic mice, knockout of PGRN, which is significantly reduced in DKD, aggravated mitochondrial dysfunction in podocytes (Zhou et al., 2019). Treatment with recombinant human PGRN promoted mitophagy and mitochondrial biogenesis, thereby alleviating mitochondrial dysfunction and podocyte injury. A potential mechanism by which PGRN protects mitochondria is mediated via PGRN–SIRT1–PGC1α regulation of FOXO1 (Zhou et al., 2019).

### Mitochondrial Biogenesis

Mitochondrial biogenesis replicates mtDNA, generates new and functional mitochondria, and increases ATP production by the proliferation of pre-existing organelles (Jiang et al., 2020). Mitochondrial biogenesis and its concomitant cellular processes enhance metabolic pathways and antioxidant defense mechanisms that mitigate injury from tissue hypoxia, excess production of ROS, and glucose or fatty acid overload, all of which contribute to the pathogenesis of kidney disease, including DKD. The process of mitochondrial biogenesis is largely regulated by networks of transcription factors that link external cues to cell energy demand and adaptive responses (Galvan et al., 2017).

Peroxisome proliferator-activated receptor γ coactivator-1α (PGC-1α) is a prominent transcriptional coactivator that interacts with other transcription factors to regulate mitochondrial biogenesis in a variety of cells including podocytes (Svensson et al., 2016; Li S.-Y. et al., 2017). PGC-1α acts as the “master regulator” in stimulating the expression of mitochondrial genes as well as nuclear genes in response to extracellular signals, energetic demand, or mitochondrial dysfunction. Several experimental models of DKD exhibit reduced efficiency of mitochondrial biogenesis, decreased PGC-1α levels, and defective mitochondrial function (Sun et al., 2014; Li S.-Y. et al., 2017; Zhang et al., 2018). The downregulation of PGC-1α and its downstream signalling cascades has been proposed to be the key contributor to renal lipid overload, mitochondrial loss and dysfunction, eventually leading to podocyte injury and destruction of the GFB (Long et al., 2016; Li S.-Y. et al., 2017; Li and Susztak 2018). Endogenous expression of PGC-1α in podocytes exhibited protective effects against kidney fibrosis in mice with DKD (Zhang et al., 2018). Furthermore, PGC-1α is negatively regulated by upstream open reading frames (uORFs) (Dumesic et al., 2019), Smad3, and NF-κB (Dai et al., 2021). PGC-1α expression is positively regulated by AMPK (Dugan et al., 2013), sirtuins (Yacoub et al., 2014), Ewing sarcoma breakpoint region 1 (EWSR1) (Park et al., 2015), PGRN (Zhou et al., 2019), G protein-coupled bile acid receptor TGR5 (Wang et al., 2016), and induced-by-high-glucose 1 (IHG-1) (Hickey et al., 2011), which subsequently activates nuclear respiratory factors (NRFs), improving mitochondrial DNA expression and protein translation and thus promoting mitochondrial biogenesis. For example, it has been demonstrated the activation of TGR5 with its agonist INT-777 can induce mitochondrial biogenesis and attenuate renal oxidative stress in db/db mice and human podocyte cell line (Wang et al., 2016).

The activation of peroxisome proliferator-activated receptors (PPARs) and oestrogen-related receptors (ERRs) is also involved in the regulation of mitochondrial biogenesis, sometimes by these receptors co-operating with PGC-1α (Fan and Evans 2015). ERRs upregulate the entire gene network necessary for biogenesis, but in contrast to ERRs, PPARs are not sufficient by themselves to fully induce biogenesis (Weinberg 2011). Numerous studies have demonstrated that the activity of PPARγ, the third member of the PPARs (PPARα, PPARβ/δ and PPARγ), is pivotal in protecting podocytes (Agrawal et al., 2021). PPARγ attenuates the renal effects of aging and generally promotes mitochondrial biogenesis by inducing PGC-1α (Hondares et al., 2006; Weinberg 2011). PGC-1α can directly bind to nuclear receptors PPARs and ERRs and coactivate the transcription of genes. PPARγ agonists (such as thiazolidinediones) have been shown to delay DKD progression in patients with type 2 diabetes mellitus and in various animal models of diabetes (Yang et al., 2012).

Furthermore, multiple other factors act directly or indirectly to regulate mitochondrial biogenesis in podocytes. Mitochondrial glycerol 3-phosphate dehydrogenase (mGPDH) is defined as a component in the respiratory chain, which guarantees the appropriate production of energy in a cell. Recently, Qu *et al.* verified that podocyte-dominated expression of mGPDH was downregulated in DKD, and activation of mGPDH induced mitochondrial biogenesis and reinforced mitochondrial function (Qu et al., 2021). The role of transcription factor EB (TFEB) as a key regulator of the autophagy-lysosome pathway has been widely investigated (Sardiello et al., 2009; Settembre et al., 2011). TFEB can regulate mitochondrial biogenesis in PGC-1α-dependent or PGC-1α-independent pathways (Kang et al., 2019; Wang S. et al., 2020). Adenosine is significantly increased in response to various cellular damages. Treatment of db/db mice with the adenosine receptor A_3_AR antagonist LJ-2698 has a renoprotective effect by modulating PGC-1α (Dorotea et al., 2018).

PGC-1α interacts with many transcription factors and is implicated in complex biological functions. Currently, there are no drugs that specifically target PGC-1α in clinical trials. It is reasonable to assume that a strategy targeting upstream or downstream molecules of PGC-1α pathway is possible. It should be noted, however, that podocytes may have a narrow PGC-1α tolerance and that excessive PGC-1α may alter mitochondrial properties. It has been proven that transgenic overexpression of PGC-1α in podocytes causes uncontrolled mitochondrial proliferation and glomerulopathy in mice (Li S.-Y. et al., 2017).

### Oxidative Phosphorylation and ROS Production

The term ROS encompasses a wide range of highly reactive, oxygen-containing molecules, including free radical species, such as hydroxyl radicals and superoxide radicals, and non-radical species, such as hydrogen peroxide. ROS are well known for their role in mitochondrial dysfunction and the development of diabetic microvascular complications, including DKD. ROS are historically considered toxic by-products of pathological cellular metabolism, but the current consensus is that ROS have physiological functions at low levels and take part in promoting the proliferation and survival of cells in response to stress. However, if ROS generation is not balanced through appropriate regulation of synthesis and degradation, oxidative stress may occur. ROS levels that exceed the antioxidant capacity are a sign of mitochondrial dysfunction and a risk factor for DKD (Dugan et al., 2013; Coughlan et al., 2016).

There are several sources of ROS in human cells, but the main endogenous ROS are generated from mitochondria via the respiratory chain (Scialo et al., 2016). Hyperglycemia is a representative hallmark of diabetes and is closely linked to excessive ROS, which is an important pathway contributing to the pathogenesis of diabetes associated complications. Under diabetic conditions, excessive glucose enters into the tricarboxylic acid cycle (TAC), which results in more NADH or FADH_2_ entering the mitochondrial electron transport chain. Under this condition, electron transfer is obstructed, and some of them escape to generate superoxide in both the intermembrane space and matrix, which results in excessive production of ROS (Brownlee 2005). It has been firmly established that HG exposure of glomerular podocytes results in an increased ROS level. Overproduction of ROS in the presence of HG induces mtDNA damage and protein and lipid peroxidation, subsequently resulting in mitochondrial dysfunction and podocyte injury. Notably, glucose-induced excessive ROS production plays a central role in initiating podocyte apoptosis and podocyte depletion followed by progression to renal damage (Susztak et al., 2006; Fakhruddin et al., 2017). Pyruvate kinase isoform M2 (PKM2) is a rate-limiting glycolytic enzyme. Qi *et al.* shows that podocyte-specific *Pkm2*-knockout in mice aggravates albuminuria and pathological severity of DKD (Qi et al., 2017). PKM2 activator (TEPP-46) can significantly ameliorate mitochondrial dysfunction by increasing glucose metabolic flux, preventing the elevation of ROS production, inducing mitochondrial biogenesis (Qi et al., 2017). In addition, treatment with antioxidants, such as Grape seed proanthocyanidin extracts (Bao et al., 2014), INO-1001 or PJ-34 (Szabo et al., 2006), has been shown to restore mitochondrial dysfunction and attenuate kidney injury in animal models of DKD.

The NADPH oxidase (Nox) family is another important endogenous source of ROS production. The mammalian Nox has seven isoforms: Nox1 to Nox5, Duox1, and Duox2. Nox4 is the predominant form within the kidney, whereas Nox1, Nox2 and Nox5 are also expressed in the kidney (Holterman et al., 2015). NOXs, particularly NOX4, have been reported to be pathologically relevant sources of ROS in HG-induced podocytes leading to mitochondrial damage and podocyte apoptosis. Furthermore, *in vivo* studies, genetic deletion of NOX4 in podocytes or treatment with a novel NOX1/4 inhibitor (GKT137831) reduced oxidative stress, podocyte injury and the development of DKD (Jha et al., 2014; Jha et al., 2016; Gray et al., 2017). Similar results were obtained using salvianolate, a prescribed Chinese medicine derived from Danshen, through regulation of NOX4 activity in db/db mice (Liang et al., 2021).

As described above, PGC-1α is considered to be a master, upstream transcriptional regulator of oxidative phosphorylation and mitochondrial biogenesis (Galvan et al., 2017) and PGC-1α levels were reduced in DKD (Sun et al., 2014; Zhang et al., 2018). Endogenous PGC-1α also exhibited protective effects against renal fibrosis in diabetic mice through an anti-oxidative mechanism (Zhang et al., 2018). Recently, a study highlighted that PGC-1α-mediated mitochondrial bioenergetics could play a pivotal role in lipid disorder-induced podocyte injury and the development of DKD, whereas restoring PGC-1α activity and a balanced energy supply via berberine may be a novel therapeutic strategy for the treatment of DKD (Qin et al., 2020).

The family of NAD^+^-dependent deacetylases known as sirtuins (SIRT1-7) has an essential role in the regulation of mitochondrial function of podocytes in DKD (Hershberger et al., 2017; Fan et al., 2019; Zhang et al., 2019). It has been reported that Sirt6 alleviates HG-induced mitochondrial damage and oxidative stress in podocytes through AMPK activation (Fan et al., 2019). Importantly, SIRT1-mediated deacetylation of PGC-1α could ameliorate HG-induced podocyte damage (Cai et al., 2016; Zhang et al., 2019) and resveratrol, an activator of SIRT1, demonstrated significant protection of mitochondrial function in diabetic mice with DKD through SIRT1/PGC-1α-regulated attenuation of mitochondrial oxidative stress (Zhang et al., 2019; Wang F. et al., 2020). Furthermore, one other group found that salidroside, an active component from Rhodiola rosea L., ameliorates diabetic nephropathy by stimulating the Sirt1/PGC-1α axis in diabetic mice (Liang et al., 2021). The above results revealed the protective role of PGC-1α in regulating mitochondrial homeostasis in podocytes and identified potential targets for the treatment of DKD.

ROS act as a master switch for activating inflammatory responses by activating multiple downstream pathways, including nucleotide leukin-rich polypeptide 3 (NLRP3), NF-κB, and Toll-like receptor (TLR). Upon stimulation, NLRP3 can form a NLRP3 inflammasome to act as a cytosolic multiprotein caspase-activating complex platform and subsequently lead to the activation of Caspase-1. Activation of Caspase-1 can lead to the maturation and release of interleukin (IL)-1β and IL-18 (Davis et al., 2011). In a type 2 diabetic model, excessive activation of NLRP3 was associated with chronic inflammation, cell death, and fibrosis. Accumulating data suggest that mitochondrial ROS could activate the NLRP3 inflammasome in glucose or advanced glycation end product stressed podocytes (Shahzad et al., 2015; Yu et al., 2019; Wu et al., 2021). Treatment of db/db mice with the NLRP3 inflammasome inhibitor (MCC950) could attenuate podocyte damage and improve kidney function by inhibiting lipid accumulation in DKD (Wu et al., 2021). Another study reported that luteolin, a natural flavonoid found in various fruits and vegetables, attenuated HG-induced podocyte damage by suppressing the NLRP3 inflammasome pathway (Yu et al., 2019).

### Mitochondrial Protein Quality Control

The mitochondrial proteome comprises approximately 1,200 proteins in humans (Rath et al., 2021). Mitochondrial function strongly relies on protein homeostasis within organelles. Mitochondria are comprised of proteins encoded by two genomes, mitochondrial and nuclear, but approximately 99% of mitochondrial proteins are encoded by the nuclear genome, and they are synthesized in the cytosol (Song et al., 2021). Hence, the synchronization of gene expression between the nucleus and mitochondria and efficient import of the encoded mitochondrial proteins into the specific locations in the mitochondria from the cytosol are essential for mitochondrial protein homeostasis. To repair or degrade misfolded and damaged proteins, mitochondria rely on several quality control pathways, including mitochondrial molecular chaperones promoting folding of misfolded proteins and ATP-dependent proteases degrading misfolded or damaged mitochondrial proteins (Vazquez-Calvo et al., 2020). Disturbances in mitochondrial protein homeostasis lead to proteotoxic insults and cell injury (Cybulsky 2017). Notably, mitochondrial protein homeostasis is challenging under HG conditions. As described above, HG exposure of podocytes leads to elevated ROS, whereas continuous intracellular ROS elevation can impair protein function and induce inflammatory responses, leading to cellular death.

Most mitochondrial proteins are synthesized in the cytosol, and then precursor proteins are bound to molecular chaperones and imported into mitochondria. Molecular chaperones are enzymes whose functions are responsible for stabilizing, folding, and unfolding precursor proteins. Heat shock proteins (HSPs) are highly conserved proteins that act as molecular chaperones and play a vital role in protein homeostasis (Young et al., 2003; Song et al., 2021). Increased levels of HSP25, HSP60, and HSP70 are observed in the diabetic outer medulla, but no differences were detected in the glomeruli in response to diabetes (Barutta et al., 2008). Only the phosphorylated form of HSP27 is increased in the podocytes of diabetic animals (Barutta et al., 2008).

The major response to excessive amounts of unfolded or misfolded proteins is activation of UPR pathway. There are two distinct UPRs—the endoplasmic reticulum unfolded protein response (UPRER) and the mitochondrial unfolded protein response (UPRmt)—that stabilize, renature, and degrade unfolded proteins in the mitochondria and the ER, respectively. However, prolonged and severe UPR can lead to an excessive ER stress and result in pro-apoptotic cell death. Although the UPRER and UPRmt involve chaperones and proteases specific to each organelle, both pathways interact and influence each other upon activation in response to extrinsic stimuli (Senft and Ronai 2015; Tang et al., 2021). Notably, the sustained UPRER pathway has been implicated in the pathogenesis of podocyte injury and DKD (Cybulsky 2017; Kang et al., 2017; Wang et al., 2021). Therefore, activation of the UPRmt might have also occurred in DKD. One study in a rat model of streptozotocin-induced diabetes showed that exposure to HG activated the UPRER pathway in renal podocytes, whereas treatment with an endogenous peptide (intermedin), which has anti-inflammatory and antioxidant properties, blocked such ER stress responses and alleviated podocyte apoptosis (Wang et al., 2021). The regulation of mitochondrial protein quality control in polypeptide sorting, folding, transportation and subsequent assembly into multiprotein complexes during mitochondrial biogenesis is essential for mitochondrial function and cellular survival. However, the functional association between the mitochondrial protein quality control system, podocyte injury and DKD remains poorly understood.

## Mitochondria-Associated Endoplasmic Reticulum Membranes

To maintain homeostasis, organelles work cooperatively (Inoue et al., 2019). Certainly, mitochondria within a cell cannot exist in isolation. They interact with other subcellular organelles, particularly with the ERs. Recently, an emerging concept is that the ER and mitochondria are organized as a complex network through direct interactions at membrane contact sites called MAMs (mitochondria associated ER membranes) (Kornmann et al., 2009). At the MAMs, the membrane of the juxtaposed ER and mitochondria are separated by only 10–25 nm. This proximity not only allows direct contact of proteins and lipids but also exchanges of Ca^2+^ in the ER with those in the OMM (Csordas et al., 2006). Perturbations in MAMs and increased ER-mitochondria contacts have been reported in various neurodegenerative disorders (Parakh and Atkin 2021) and metabolic disorders (Yang et al., 2020), as well as DKD (Yang et al., 2021). The disturbance of MAMs leads to abnormal intracellular Ca^2+^ levels, mitochondrial damage, ER stress, autophagy, and apoptosis (Inoue et al., 2019).

Calcium signalling plays a vital role in many cellular physiological pathways. In pathological states, calcium signals can precipitate mitochondrial damage and trigger cell death, particularly when accompanied by energetic deprivation and oxidative stress (Bhosale et al., 2015). Therefore, the role of mitochondria as sensors and modulators of calcium signalling is extremely important. An increase in the inflow of Ca^2+^ into mitochondria from the juxtaposed ER takes place through this specialized proximity during various common stresses. An appropriate elevation in Ca^2+^ concentration activates TAC dehydrogenase to promote ATP production. However, an excessive elevation in Ca^2+^ concentration opens the mitochondrial permeability transition pore and releases cytochrome c simultaneously, leading to cellular apoptosis (Inoue et al., 2019). In the unilateral ureteral obstruction model, mitochondria and the ER form a pathological feedback loop by Ca^2+^ dysregulation and ER stress pathways, resulting in the impairment of both organelles (Martinez-Klimova et al., 2020). Ca^2+^ channel transient receptor potential cation channel subfamily V member 1 (TRPV1), a channel modulating the intracellular Ca^2+^ concentration, can be activated by multiple endogenous stimuli, including pressure, force, and exogenous stimuli, such as capsaicin (Nieto-Posadas et al., 2011). Activation of the TRPV1 channel by capsaicin can play a robust therapeutic role in HG-induced mitochondrial damage in podocytes, accompanied by decreased MAM formation and reduced Ca^2+^ transport from the ER to mitochondria (Wei et al., 2020).

As described above, mitophagy refers to a protective effect in response to diverse stimuli, including hypoxia, ROS, and energy stresses (Tang et al., 2020). ER-mitochondria contact sites are essential for organelle quality control by involving in mitophagy (Hamasaki et al., 2013). Researchers have demonstrated that many proteins involved in mitophagy are recruited to MAMs following mitophagic stimuli, in turn, recruited autophagy-associated proteins promote the formation of MAMs and autophagosomes (Yao et al., 2021). Currently, the FUNDC1-mediated pathway is one of the well-studied pathways of the PINK1/Parkin-independent mitophagy as described above. FUNDC1 has been proven to accumulate at MAMs, which can initiate mitochondrial division prior to mitophagy (Wu et al., 2016). FUNDC1-dependent mitophagy plays a protective role in acute reperfusion injury and chronic metabolic syndrome via its sustaining mitochondrial homeostasis activity. As shown in proximal tubule-specific Fundc1 knockout mice, ischemic preconditioning activates FUNDC1-dependent mitophagy, and FUNDC1 deficiency abolishes the benefits of ischemic preconditioning against renal ischemia reperfusion injury. Mechanistically, FUNDC1 deficiency provoked compromised mitochondrial quality control, manifested by abnormal mitochondrial quality and excessive Drp1-dependent mitochondrial fission (Wang J. et al., 2020). Although more studies on the role of MAMs in mitophagy in DKD are needed, the available evidence suggests that MAMs provide a platform for autophagy-associated proteins to perform their biological functions.

Mitochondria are highly plastic organelles that undergo fission and fusion to optimize their function (Youle and van der Bliek 2012). Disturbances of mitochondrial dynamics, featuring excessive mitochondrial fission, are noted in glomerular podocytes in diabetic nephropathy (Ni et al., 2017; Ma et al., 2019; Qin et al., 2019). As described previously, mitofusin 2 (Mfn2) is enriched at the ER-mitochondria interface and plays a key role in the maintenance of mitochondrial fusion and fission (de Brito and Scorrano 2008). MAMs are involved in the early steps of mitochondrial fission by marking the division sites (Csordas et al., 1999). Despite these interesting findings, the precise role and regulation of MAMs in the development and progression of DKD await further investigation.

## Targeting Mitochondrial Dysfunction

Although the precise roles of mitochondrial function in podocyte health and disease are not completely understood, targeting mitochondria could be a very promising strategy to treat podocyte dysfunction. Various therapeutic strategies that target mitochondria are under investigation for the treatment of podocyte dysfunction and/or DKD. Emerging data from preclinical and preliminary clinical data suggest that targeting mitochondrial dysfunction is a sound rationale. Several investigational drugs are in different stages of clinical evaluation. Although some of these drugs are currently used in clinical trials for the treatment of other CKDs, preclinical data suggest that these therapies are also promising agents for the treatment of DKD by targeting the mitochondrial function of podocytes.

Antioxidants are the oldest class of drugs used to counteract ROS generation and treat mitochondrial dysfunction. Currently, the majority of ongoing clinical trials for the treatment of mitochondrial diseases are still based on the use of antioxidants (Forbes and Thorburn 2018). In mitochondria, Coenzyme Q10 (CoQ10; ubiquinone) is a pivotal component of the mitochondrial respiratory chain with powerful antioxidant capacity, as it shuttles electrons from both complexes I and II to complex III of the electron transport chain. Knockout of genes involved in coding the CoQ10 biosynthesis pathway enzymes in glomerular podocytes is sufficient to induce the typical phenotypes of nephrotic syndrome and focal segmental glomerular sclerosis (Widmeier et al., 2019; Widmeier et al., 2020). CoQ10 therapy of rodent models with DKD could significantly decrease albuminuria and prevent detrimental changes in mitochondrial function, indicating its potent protective effect on the GFB and podocytes (Sourris et al., 2012). Case reports and case series have reported the treatment with CoQ10 or its synthetic analogue idebenone significantly reduced albuminuria in paediatric patients with COQ6 glomerulopathy or ADCK4 mutation (Feng et al., 2017; Stanczyk et al., 2018). CoQ10 is not water soluble, which limits its transport to the IMM. More recently, more soluble and hydrophilic 2,4-dihydroxybenzoic acid (2,4-diHB) has been shown to have a strong effect in rescuing podocyte function and preventing renal disease caused by primary dysfunction in the CoQ10 biosynthesis pathway (Widmeier et al., 2019; Widmeier et al., 2020). These findings warrant further evaluation in prospective human studies in the near future. MicroRNA-21 (miR-21) has been widely studied in kidney disease because of its important antiapoptotic effects (Chen et al., 2018; Wang et al., 2019). MiR-21 expression is upregulated in kidney tissues of DKD patients and HG-treated podocytes, and the down-regulation of miR-21 inhibited the progression of DKD (Chen et al., 2018). Furthermore, miR-21 is involved in the regulation of mitochondrial dysfunction by disrupting ROS homeostasis (La Sala et al., 2018). Lademirsen is an antisense oligonucleotide that inhibits miR-21, and there is some evidence that it can enhance mitochondrial function in podocytes; it is currently under clinical evaluation in Alport syndrome patients (NCT02855268) (Gomez et al., 2015). Nuclear respiratory factor 2 (Nrf2), a master regulator of the stress response, is relatively inactive under non-stressed conditions. Activation of Nrf2 inhibits the expression of Drp1 and mitochondrial fission, leading to enhanced mitochondrial fusion and survival (Zhu et al., 2019). Drp1 hyperactivation and excessive/pathological mitochondrial fission occur in various DKD models, and selective activation of Nrf2 is sufficient for its anti-senescent and podocyte protective effects (Fang et al., 2021). Due to this, its potential as a therapeutic target in DKD has been increasingly discussed. Bardoxolone methyl is a novel, small-molecule inhibitor of Nrf2 that improves kidney function in several glomerular diseases and is under clinical evaluation in patients with chronic kidney disease (NCT03749447) or DKD (NCT03550443) (Zhou et al., 2020; Daehn and Duffield 2021) (Table 1).

**TABLE 1:** Potential approaches to target podocyte mitochondrial dysfunction in clinical studies.

Agent	Mechanism of action	*In vivo* and clinical studies	References
Coenzyme Q10	Antioxidant	1) Decreases albuminuria and prevents detrimental changes in mitochondrial function rodent models with DKD	Sourris et al. (2012), Stanczyk et al. (2018), Feng et al. (2017)
		2) Reduces albuminuria in paediatric patients with COQ6 glomerulopathy or ADCK4 mutation	
Lademirsen	Inhibits microRNA-21	1) Down-regulation of miR-21 inhibits the progression of DKD in streptozotocin- induced diabetic nephropathy rats	Gomez et al. (2015); Chen et al. 2018)
		2) Phase II study (NCT02855268) in patients with Alport syndrome	
Bardoxolone methyl	Activates of Nrf2 and inhibits the expression of Drp1 and mitochondrial fission	1) Decreased albuminuria and has a renoprotective role for podocytes and diabetic glomerulopathy in diabetic nephropathy mice	Fang et al. (2021), Zhou et al. (2020)
		2) Phase III study (NCT03550443) in patients with diabetic kidney disease	

In addition, although several previous and current drugs to treat DKD do not directly target mitochondrial function, they play a protective role under conditions that affect mitochondria. For example, the Study of Diabetic Nephropathy with Antrasentan (SONAR) trial found that the endothelin A receptor (EAR) antagonist atrasentan reduced the risk of renal events by 35% in patients with DKD (Heerspink et al., 2019). Analysis of urinary metabolites from DKD patients treated with atrasentan revealed that it might prevent the progression of mitochondrial dysfunction (Pena et al., 2017). Similar results were obtained in DKD patients treated with sodium glucose cotransporter 2 (SGLT2) inhibitors, such as dapagliflozin, canagliflozin, and empagliflozin (Liu et al., 2020; Liu et al., 2021). Currently, there are no studies investigating the effect of EAR antagonists or SGLT2 inhibitors on the mitochondrial function of podocytes. This may be worthy of further study.

Despite the fact that direct targeting of mitochondrial function as a therapeutic approach is not satisfactory, the development of specific molecules targeting mitochondria and mitochondria-associated signalling pathways for therapeutic gain is a rapidly evolving field. In recent years, numerous specific molecules and treatment strategies targeting various aspects of mitochondrial dysfunction have been reported. The targets involve many genes controlling mitochondrial biogenesis and energy homeostasis, including PGC-1α, Drp1 and ROS. The molecular targets, mechanisms of action and therapeutic effects of these preclinical drugs are summarized in Table 2.

**TABLE 2:** Potential approaches to target podocyte mitochondrial dysfunction in preclinical developments.

Agent	Classification	Mechanism of action	DKD model	References
Mitochondria-targeted antioxidant	Salvianolate	Modulates NOX4 activity and ameliorates oxidative injury	Db/db mice and human podocyte cell line	Liang et al. (2021)
	MCC950	Inhibits NLRP3 inflammasome and suppresses lipid accumulation, ROS generation and NF-κB p65 activation	Db/db mice and mouse podocyte cell line	Wu et al. (2021)
	Berberine	Activates the PGC-1α signalling pathway and promotes mitochondrial fatty acid oxidation	Db/db mice and mouse podocyte cell line	Qin et al. (2020)
	Resveratrol	Activates SIRT1 and suppresses oxidative stress	Db/db mice and human podocyte cell line	Wang et al. (2020a), Zhang et al. (2019)
	GKT137831	Inhibits Nox1/4 activity and suppresses ROS generation	Streptozotocin-induced diabetic mice and human podocyte cell line	Jha et al. (2014)
	INO-1001 and PJ-34	Inhibits poly (ADP-ribose) polymerase activity and blocks the ROS generation	Db/db mice and mouse podocyte cell line	Szabo et al. (2006)
	Grape seed proanthocyanidin extracts	Activates the AMPK-SIRT1-PGC-1a signaling pathway and inhibits oxidative stress	Streptozotocin-induced diabetic mice	Bao et al. (2014)
Inhibits mitochondrial fission	Mdivi-1	Inhibits DRP1 activity and suppresses mitochondrial fission	Db/db mice and primary mouse podocyte	Ayanga et al. (2016)
	Berberine	Inhibits palmitic acid-induced activation of DRP1 activity and suppresses mitochondrial fission	Db/db mice and mouse podocyte cell line	Qin et al. (2019)
Promotes mitochondrial biogenesis	LJ-2698	Inhibits adenosine receptor activity and promotes mitochondrial biogenesis	Db/db mice	Dorotea et al. (2018)
	Salidroside	Stimulates the Sirt1/PGC-1 axis and promotes mitochondrial biogenesis	Streptozotocin-induced diabetic mice	Xue et al. (2019)
	TEPP-46	Activates pyruvate kinase M2 and induces mitochondrial biogenesis	Streptozotocin-induced diabetic mice, mouse and human podocyte cell lines	Qi et al. (2017)
	INT-777	Activates G protein-coupled receptor TGR5 and induces mitochondrial biogenesis	Db/db mice and human podocyte cell line	Wang et al. (2016)

None of the existing drugs could be specifically targeted to mitochondrial health of podocytes. Nevertheless, a success in one of these ongoing clinical trials will provide important new insights into the development of innovative regimens for podocyte dysfunction. Therefore, there is a continuing need to identify novel and more specific targets to target mitochondria to widen the scope of current treatments for DKD. In addition to identifying effective therapeutic agents, considering the optimal timing for these interventions is also needed. Predictive biomarkers that can help guide decision-making to target mitochondrial dysfunction to prevent DKD are likewise lacking.

## Future Directions

Although increasing amounts of evidence has demonstrated that mitochondrial dysfunction of podocytes is involved in the development and progression of DKD, our understanding of the role of mitochondrial damage in DKD remains limited. There are several unanswered questions in this area. First, it is clear that mitochondrial dysfunction is a common pathological hallmark and occurs early in DKD. Although much is known about mitochondrial dynamics, mitophagy, ROS production and biogenesis, the exact role and interaction of each process in DKD remains unclear. Second, despite evidence that MAMs play an important role during the development and progression of DKD, their precise role remains largely unclear. The relationship between the MAMs and mitochondrial dysfunction has attracted increasing attention. A further understanding of the role of the MAMs in mediating mitochondrial dysfunction under hyperglycemic condition might lead to new therapeutic options for DKD. Third, there are currently no mitochondria-targeting therapeutic agents approved for DKD. Modern small-molecule drug design, advances in nucleic acid-based therapeutics, and novel nano-drug delivery systems have greatly assisted in enhancing bioavailablity and mitochondrial targeting during the development of more effective therapeutic agents. We can therefore expect many more innovations to occur in the near future.
